# The Natural Cytotoxicity Receptor NKp44 (NCR2, CD336) Is Expressed on the Majority of Porcine NK Cells *Ex Vivo* Without Stimulation

**DOI:** 10.3389/fimmu.2022.767530

**Published:** 2022-01-28

**Authors:** Kerstin H. Mair, Assiatu J. Crossman, Bettina Wagner, Susanna Babasyan, Leela Noronha, Patricia Boyd, Dante Zarlenga, Maria Stadler, Katinka A. van Dongen, Wilhelm Gerner, Armin Saalmüller, Joan K. Lunney

**Affiliations:** ^1^ Institute of Immunology, Department of Pathobiology, University of Veterinary Medicine Vienna, Vienna, Austria; ^2^ CD Laboratory for Optimized Prediction of Vaccination Success in Pigs, Institute of Immunology, Department of Pathobiology, University of Veterinary Medicine Vienna, Vienna, Austria; ^3^ Animal Parasitic Disease Laboratory, Beltsville Agricultural Research Center (BARC) Agricultural Research Service (ARS), United States Department of Agriculture (USDA), Beltsville, MD, United States; ^4^ Center for Cancer Research, National Cancer Institute, National Institutes of Health (NIH), Bethesda, MD, United States; ^5^ Department of Population Medicine and Diagnostic Sciences, College of Veterinary Medicine, Cornell University, Ithaca, NY, United States; ^6^ United States Department of Agriculture (USDA) Agricultural Research Service (ARS) Arthropod-Borne Animal Diseases Research Unit, Agricultural Research Service (ARS), United States Department of Agriculture (USDA), Manhattan, KS, United States; ^7^ The Pirbright Institute, Woking, United Kingdom

**Keywords:** NKp44, NK cells, CD336, swine, activation marker, monoclonal antibody

## Abstract

Natural killer (NK) cells have been studied extensively in humans and mice for their vital role in the vertebrate innate immune system. They are known to rapidly eliminate tumors or virus infected cells in an immune response utilizing their lytic properties. The natural cytotoxicity receptors (NCRs) NKp30 (NCR3), NKp44 (NCR2), and NKp46 (NCR1) are important mediators of NK-cell cytotoxicity. NKp44 expression was reported for NK cells in humans as well as in some non-human primates and found exclusively on activated NK cells. Previously, no information was available on NKp44 protein expression and its role in porcine lymphocytes due to the lack of species-specific monoclonal antibodies (mAbs). For this study, porcine-specific anti-NKp44 mAbs were generated and their reactivity was tested on blood and tissue derived NK cells in pigs of different age classes. Interestingly, NKp44 expression was detected *ex vivo* already on resting NK cells; moreover, the frequency of NKp44^+^ NK cells was higher than that of NKp46^+^ NK cells in most animals analyzed. Upon *in vitro* stimulation with IL-2 or IL-15, the frequency of NKp44^+^ NK cells, as well as the intensity of NKp44 expression at the single cell level, were increased. Since little is known about swine NK cells, the generation of a mAb (clone 54-1) against NKp44 will greatly aid in elucidating the mechanisms underlying the differentiation, functionality, and activation of porcine NK cells.

## Introduction

Natural killer (NK) cells are specialized innate effector lymphocytes identified by their potent cytolytic activity against tumor or virus infected cells (1). NK cells express different activating and inhibitory receptors on their surface; the balance of their signaling mediates the functional outcome when the NK cell encounters a potential target cell (2). One important family of activating receptors are the natural cytotoxicity receptors (NCRs) comprised of three members, NKp30 (NCR1, CD337), NKp44 (NCR2, CD336) and NKp46 (NCR3, CD335), that play important roles in NK-cell recognition of aberrant or infected cells (3). NKp44 was extensively studied on human NK cells but to date, there is no clear evidence of NKp44 expression in the mouse (4, 5). Nonetheless, NKp44 transcripts were found in non-human primates (6). While five-fold higher NKp44 transcript levels were found in chimpanzees, much lower differential increases were observed upon cell activation when compared with humans. In *Macaca fascicularis*, NKp44 was found at the transcript level, but due to frame shifts in the sequence, only abortive transcripts were detected (6).

NKp44 is a 44 kDa transmembrane glycoprotein characterized by a single extracellular V-type domain. The transmembrane domain contains the charged amino acid lysine and is involved in the association of NKp44 with the adaptor protein DAP12 containing an ITAM (immunoreceptor tyrosine-based activation motif) for signaling (4, 7, 8). Crosslinking of NKp44 leads to activation of NK cells and release of cytokines like IFN-γ and TNF-α as well as target cell lysis (7). Additionally, NKp44 contains an ITIM (immunoreceptor tyrosine-based inhibitory motif), in its cytoplasmic domain, indicating inhibitory functions and therefore dual functionality of the receptor (4). In contrast to NKp30 and NKp46, NKp44 is not constitutively expressed on resting human NK cells, but can be induced *in vitro* by cytokines like IL-2 and IL-15 (7, 9, 10). Therefore, NKp44 can be seen as an activation marker for human NK cells. Reduced levels of NKp44 correlate with a decreased function of NK cells, e.g., in HIV patients (11).

Different ligands have been described for NKp44, e.g., in the recognition and killing of various tumor cell lines (12). Surface heparan sulfate proteoglycans and platelet-derived growth factor expressed by tumor cells are involved in target cell recognition, NKp44-mediated degranulation as well as IFN-γ and TNF-α secretion (13–15). Similar to NKp46, NKp44 can bind the hemagglutinins of influenza and Sendai viruses resulting in functional activation of NK cell cytotoxicity and IFN-γ production (16, 17). NKp44 recognizes envelope proteins of West Nile and Dengue viruses (18). Furthermore, Esin et al. showed that NKp44 was able to bind to the surface of different species within the *Mycobacterium* genus and *Pseudomonas aeruginosa* (19, 20). An inhibitory function for NKp44 was presented by Rosental et al. (21), demonstrating that binding of NKp44 to proliferating cell nuclear antigen (PCNA), that is overexpressed in some cancer cells, mediates NKp44/ITIM signaling, thus promoting cancer survival.

In humans, NKp44 is not found exclusively on NK cells. NKp44 expression has also been observed on a subset of plasmacytoid dendritic cells (pDCs) in human tonsils *ex vivo* and can be induced in pDCs in blood after IL-3 stimulation. Nevertheless, NKp44 triggering inhibits IFN-α production in CpG stimulated pDCs (22, 23). A subset of human γδ T cells can express NKp44 upon IL-15 stimulation *in vitro* and these cells showed NKp44-dependent cytotoxic activity against myeloma target cell lines (24). Additionally, NKp44 molecules are expressed on innate lymphoid cells as they are found on RORγt^+^ IL-22 producing cells (25, 26).

Unlike human and mouse NK cells, knowledge of porcine NK cells is scarce. Different groups have shown that porcine NK cells play important roles in the defense against viral and parasitic pathogens (27). Porcine NK cells were initially identified as perforin^+^CD2^+^CD3^-^CD4^-^CD5^-^CD6^-^CD8α^+^CD8β^-^CD11b^+^CD16^+^ lymphocytes (28). Recently, anti-porcine NKp46-specific mAbs were developed, leading to a better characterization of porcine NK cells. In contrast to other mammalian species, not all porcine NK cells express NKp46 (29). It was shown that porcine NK cells can be divided in three different subsets according to their NKp46 expression pattern: NKp46^-^, NKp46^+^ and NKp46^high^, and exhibit different functional properties (30). To date, NKp44 was only investigated on a transcriptional level in the pig. NKp44 transcripts were found in porcine NK cells as well as CD3^+^NKp46^+^ non-conventional T cells in the pig (31).

Here, we study NKp44 expression on the protein level after the development of an anti-porcine NKp44 mAb (clone 54-1). NKp44 expression was confirmed in cells from blood as well as lymphatic and non-lymphatic organs in pigs of different ages. In contrast to humans, we demonstrate that porcine NKp44 is expressed on resting NK cells, *ex vivo* at high levels, and can be further upregulated upon stimulation by IL-2 and IL-15. The newly developed anti-porcine NKp44 mAb (clone 54-1) will enable a comprehensive investigation into the phenotype, differentiation and functionality of porcine NK cells, and their role in swine immunity.

## Material and Methods

### Cloning and Expression of NKp44 for mAb Production

The extra cellular region of porcine anti-NKp44 (NCR2) minus the signal peptide (0-482 bp) was amplified from a pool of DNase treated cDNA from *Toxoplasma gondii* infected pig cells at BARC. The product was run on an 2% NuSieve gel that was lightly stained with ethidium bromide; the correct size band was cut out and DNA eluted and subcloned into a topo vector. After bacterial transformation clones were selected and PCR amplified and then sequenced. The resulting sequence was submitted to NCBI Gene [HQ910553 base pairs 290-808]. The plasmid was shipped from the Lunney lab to the Wagner lab at Cornell University where the NCR2 insert was recloned into the equine IL-4 vector and expressed as a fusion protein with equine IL-4 as previously described (32). After generation of stable transfectants of Chinese hamster ovary (CHO) cells the rPoNCR2/IL-4 fusion protein was purified from serum-free cell culture supernatants using an anti-IL-4 affinity column and an ÄKTA Fast Protein Liquid Chromatography instrument (GE Healthcare, Piscataway, NJ, USA) (32).

### Monoclonal Antibody Development, Screening, and Purification

BALB/c mice were immunized with the recombinant rPoNCR2/IL-4 fusion protein. The immunization, cell fusion and hybridoma selection process were performed according to a previously published protocol (33). In brief: BALB/c mice were immunized with 58 μg of the recombinant rPoNCR2 for the first injection and 24-29 μg rPoNCR2/IL-4 for all booster injections. ELISA confirmed the increase of anti-rPoNCR2 mAbs in the mouse serum. Murine spleen cells were fused to X63-Ag8.653 myeloma cells (34) and plated into 24-well plates. After 2 weeks, supernatants were screened for anti-NKp44 mAbs by direct ELISA as described earlier (33); single positive cell clones were picked, transferred into individual wells and grown up. Supernatants were frequently tested for mAb production; 8-10 weeks after fusion, cell cultures were tested for clonality by murine isotype ELISA (Sigma, St. Louis, MO). MAbs of murine IgG1 isotype were selected. Further analysis revealed these clones were positive on the rporNCR2/IL-4 transfectants by intracellular staining and flow cytometric analysis. At the same time, selected clones were tested against unrelated IL-4 fusion protein transfectants all of which were negative.

To affirm porcine cell reactivity, the anti-NKp44 supernatants were sent to BARC and screened for reactivity on pig peripheral blood mononuclear cells (PBMCs), mesenteric lymph node cells, and splenocytes using anti-NKp46 from Austria as positive control. This screen confirmed the potential reactivity of three anti-NKp44 clones (54-1, 62-1 and 62-2) out of the nine promising clones. To affirm NK cell reactivity, an immunostaining study was done comparing stimulated versus non-stimulated PBMCs. In brief, PBMCs were cultured at 4 x 10^6^ cells/ml in medium only (control cells) or with 50ng/mL recombinant porcine IL-2 (rPoIL-2, Prospect, Lot# 907RPIL201) or 50 ng/ml rPoIL-15 (Kingfisher Biotech, Lot #RP0045S-025) in 6-well plates. After 72 hours, only suspended cells were harvested, depleting samples of adherent macrophages, washed twice and resuspended in blast media at a concentration of 8.9 x 10^6^ cells/ml (cell viability 76.7%). Cells were immunostained with anti-NKp44 clone 54-1, 62-1, and 62-2, and anti-NKp46. A Goat anti-mouse IgG1-RPE was used as a secondary antibody (Southern Biotech). The stained cells (100,000 events per sample) were analyzed using the BD FACSArray. Data Analysis was performed using FlowJo v10.0.7. In a separate experiment, a two-color staining confirmed the co-expression of anti-NKp44 and CD8α^+^CD3^−^cells (data not shown).

For purification, hybridoma clone 54-1 was adapted to serum-free medium (SFM). The transfer was performed slowly by reducing the amount of FCS by 50% with each cell passage. After adaptation to SFM, clone 54-1 was grown in larger scale and mAbs were purified from supernatant using Thermo Scientific Protein A/G Agarose (Thermo Fisher Scientific Waltham, MA, #20422).

### Proof of Antigen Specificity of Anti-NKp44 Clone 54-1

RNA prepared from lung lymphocytes was used for the production of cDNA with SuperScriptTM First-Strand Synthesis System (Thermo Fisher Scientific, Vienna, Austria). Whole NKp44 coding sequence lacking the N-terminal part with the signal sequence (334-1280 nt, NCBI accession number XM_021098670.1) was amplified by S7 Fusion Polymerase (Biozym, Hessisch Oldendorf, Germany) according to manufacturer’s instructions with gene-specific primers modified with restriction overhangs for EcoRI and EcoRV (FWD: 5’-GAATTCGCTCTCAAAGGCTCATCAT CTTC-3’, REV: 5’-GATATCGAAATCAGCGACCCGGCAGC-3’, restriction sites underlined). The PCR product was cloned first into the pJET1.2 vector by blunt-end cloning for amplification in *E. coli* (CloneJET PCR cloning kit, Thermo Fisher Scientific) and subsequently cloned into the mammalian expression vector pSF-CMV-NH2-PPT-3xFLAG containing a N-terminal FLAGtag (Sigma-Aldrich, Vienna, Austria) by sticky-end cloning *via* the restriction sites. HEK293T cells were cultivated in Dulbecco’s modified Eagle’s medium (DMEM) with stable glutamine (PAN-Biotech, Aidenbach, Germany), supplemented with 10% (v/v) FCS (Sigma-Aldrich), 1 mM sodium pyruvate, 100 IU/ml penicillin, and 0.1 mg/ml streptomycin (all PAN-Biotech). HEK293T cells were transiently transfected with the expression construct using PolyFect^®^ Transfection Reagent (Qiagen, Hilden, Germany) according to manufacturer’s instructions in a 25 cm^2^ flask. After 24 hours, cells were tested in flow cytometry (FCM) as described below [see *Flow Cytometry (FCM) and Antibodies*].

### Isolation of Porcine Lymphocytes

Blood and organs were obtained from healthy 9-12 week old piglets and 2-4.5 year old sows. Animals were housed at the University Clinic for Swine at the University of Veterinary Medicine Vienna, Austria. Additionally, blood was collected from 6-month old finishing pigs from an abattoir. Animals from the slaughterhouse were subjected to electric high-voltage anaesthesia followed by exsanguination, a procedure that is in accordance to the Austrian Animal Welfare Slaughter Regulation. In-house pigs were anaesthetized by intramuscular injection of Ketaminhydrochlorid (Narketan^®^, Vétoquinol, Vienna, Austria, 10 mg/ kg body weight) and Azaperon (Stresnil^®^, Janssen Pharmaceutica, Beerse, Belgium, 1.3 mg/ kg body weight). Subsequently, animals were euthanized *via* intracardial injection of T61^®^ (MSD Animal Health, Vienna, Austria, 1.0 ml/ 10 kg body weight). This procedure was approved by the institutional ethics committee and the national authority according to § 26 of Law for Animal experiments, Tierversuchsgesetz 2012 – TVG 2012 (reference number: bmwf GZ68.205/0138-WF/V/3b/2015). All animals used for sample collection were clinically healthy and no pathological indications were observed at necropsy.

PBMC were isolated from heparinized blood using density gradient centrifugation (Pancoll human, density: 1.077 g/ml, PAN-Biotech). Dissected spleens as well as bronchial lymph nodes and tonsils were cut into small pieces and mechanically dissociated by a sieve. Spleen cells were applied to density gradient centrifugation. Isolated cells from lymph nodes and tonsils were applied to cotton-wool filtration to remove dead cells. Lymphocytes from lung tissue were isolated as described elsewhere (35). Briefly, a piece of the caudal (diaphragmatic) lung lobe was selected and the obtained lung tissue was cut in small pieces. Tissue pieces were incubated for one hour at 37°C in cell culture medium containing 2% FCS (Sigma-Aldrich), 20 mM Hepes (Sigma-Aldrich), 25 U/ml DNase I (Thermo Fisher Scientific) and 300 U/ml collagenase type I (Thermo Fisher Scientific). The obtained cell suspension was subsequently applied to cotton-wool filtration and density gradient centrifugation. Lymphocytes isolated from organs and corresponding PBMC were immediately used for phenotypic analyses by FCM. PBMC of additional piglets, sows and all 6-month old finishing pigs were frozen and stored at -150°C for further analyses.

### Isolation of Human Lymphocytes

Blood was obtained from healthy adult (18-60 years) blood donors (kindly provided by the Austrian Red Cross). PBMC were isolated from heparinized blood using density gradient centrifugation (Pancoll human, density: 1.077 g/ml, PAN-Biotech). PBMC were frozen and stored at -150°C for further analyses.

### Stimulation of Porcine and Human Lymphocytes for NKp44 Induction

For stimulation, PBMC were cultured at 2 x 10^5^ cells per well in 96-well round-bottom in RPMI 1640 with stable glutamine (PAN-Biotech) supplemented with 10% (v/v) heat-inactivated FCS (Sigma-Aldrich), 100 IU/ml penicillin, and 0.1 mg/ml streptomycin (PAN-Biotech) in a total volume of 200 µl. For later studies, porcine cells were cultured for 7 days in the presence of either 50 ng/ml rPoIL-2 (R&D Systems, Minneapolis, MN, USA) or in the presence of 50 ng/ml rPoIL-15 (Kingfisher Biotech, Saint Paul, MN, USA). Human cells were cultured for 7 days in the presence of either 50 ng/ml rhIL-2 (Roche, Vienna, Austria) or in the presence of 50 ng/ml rhIL-15 (Kingfisher Biotech). Cells cultured in medium alone served as negative control. After 7 days, cells were harvested and forwarded to FCM analyses.

### Stimulation of Porcine NK Cells by Receptor Triggering

Receptor triggering was performed by using mAbs against NKp44 (mouse IgG1, clone 54-1, in-house production) or NKp46 (mouse IgG1, clone VIV-KM3, in-house production). Isotype-matched irrelevant antibodies (clone NCG01, Dianova, Hamburg, Germany) served as control. 96-well round-bottom plates were coated with mAbs at a concentration of 3 µg/ml in PBS (50 µl per well) overnight at 4°C. Plates were washed three times with PBS prior to addition of 3 x 10^5^ cells in a total volume of 200 µl per well. Cells were pre-activated before applying them to the receptor triggering assays. For the CD107a degranulation assay, cells were pre-activated with a combination of rPoIL-2 (10 ng/ml, R&D Systems) and rPoIL-15 (10 ng/ml, Kingfisher Biotech) for 24 h. For the IFN-γ assay, cells were pre-activated with a combination of rPoIL-2 (20 ng/ml, R&D Systems) and rPoIL-18 (20 ng/ml, R&D Systems). After transfer of cells to the mAb-coated plates, cells were cultured for four hours before analyzing them by flow cytometry [see *Flow Cytometry (FCM) and Antibodies*]. For the CD107a assay, Brefeldin A (GolgiPlug, BD Biosciences, San Jose, CA, USA) and Monensin (GolgiStop, BD Biosciences) at final concentrations of 1 µg/ml and 2 µg/ml, respectively, were added to microcultures alongside the anti-CD107a mAbs for the duration of this four hours. For intracellular cytokine staining, Brefeldin A (GolgiPlug, BD Biosciences) was added to microcultures at a final concentration of 1 µg/ml for the duration of this four hours. Antibodies and reagents used for flow cytometry (FCM) staining are outlined in **Table 1**.

**Table 1:** Primary antibodies and secondary reagents used for FCM analyses.

Antigen	Clone	Isotype	Fluorochrome	Labeling strategy	Source of primary Ab
***Ex vivo* phenotyping and NKp44 induction on NK cells**					
*Porcine cells*					
CD3	BB23-8E6-8C8	IgG2a	PerCP-Cy5.5	directly conjugated	BD Biosciences
CD8α	11/295/33	IgG2a	BV421	Biotin-Streptavidin^1^	in-house
CD16	G7	IgG1	FITC	directly conjugated	Bio-Rad^2^
NKp44	54-1	IgG1	PE	secondary antibody^3^	in-house
NKp46	VIV-KM1	IgG1	Alexa647	directly conjugated	in-house
*Human cells*					
CD3	UCHT1	IgG1	BV421	directly conjugated	BioLegend
NKp44	P44-8	IgG1	PE	directly conjugated	BioLegend
NKp46	9E2	IgG1	Alexa647	directly conjugated	BioLegend
***Ex vivo* phenotyping and NKp44 induction on T cells**					
*Porcine cells*					
CD3	BB23-8E6-8C8	IgG2a	PerCP-Cy5.5	directly conjugated	BD Biosciences
CD4	74-12-4	IgG2b	BV421	secondary antibody^4^	in-house
CD8β	PPT23	IgG1	Alexa488	directly conjugated	in-house
TCRγδ	PPT16	IgG2b	Alexa 647	directly conjugated	in-house
NKp44	54-1	IgG1	PE	secondary antibody^3^	in-house
**Receptor triggering with plate-bound antibodies**					
*CD107a assay*					
CD3	BB23-8E6-8C8	IgG2a	PerCP-Cy5.5	directly conjugated	BD Biosciences
CD8α	295/33-25	IgG2a	PE	directly conjugates	BD Biosciences
CD16	G7	IgG1	FITC	directly conjugated	Bio-Rad
CD107a	4E9/11	IgG1	Alexa647	directly conjugated	Bio-Rad
*IFN-γ production*					
CD3	BB23-8E6-8C8	IgG2a	PerCP-Cy5.5	directly conjugated	BD Biosciences
CD8α	295/33-25	IgG2a	PE	directly conjugates	BD Biosciences
CD16	G7	IgG1	FITC	directly conjugated	Bio-Rad
IFN-γ	CC302	IgG1	Alexa647	directly conjugated	Bio-Rad

^1^Streptavidin-BV421, BioLegend, San Jose, CA, USA.

^2^Bio-Rad, Hercules, CA, USA.

^3^goat anti-mouse IgG1-PE, Southern Biotech, Birmingham, AL, USA.

^4^goat anti-mouse IgG2b-BV421, Jackson ImmunoResearch.

### Flow Cytometry (FCM) and Antibodies

For FCM analyses in Vienna, cells were re-suspended in PBS (PAN-Biotech) containing 10% (v/v) porcine plasma (in-house preparation) for porcine cells or in PBS containing 3% (v/v) FCS for human PBMC, HEK293T cells and cultured porcine PBMC. All incubation steps were performed in 96-well round-bottom plates at 4°C for 20 min. Primary mAbs as well as secondary reagents used for each assay are listed in **Table 1**. Non-commercial antibodies other than anti-porcine NKp44 were produced in-house at the Institute of Immunology, University of Veterinary Medicine Vienna, Austria (36). Where indicated, these antibodies were conjugated either to Alexa Fluor-647 fluorochromes (Labeling Kit, Thermo Fisher Scientific) or biotin (Sulfo-NHS-LC Biotin, Thermo Fisher Scientific) according to manufacturers’ protocols. For the use of unlabeled and directly conjugated antibodies with the same isotype a sequential staining was performed. After labeling with unconjugated primary mAb and isotype-specific dye-conjugated secondary antibodies, free binding sites were blocked by whole mouse IgG (2 µg per sample, Jackson ImmunoResearch, Suffolk, UK). Thereafter, cells were incubated with directly labeled primary mAbs. For exclusion of dead cells, Fixable Near-IR Dead Cell Stain Kit or viability dye VDeFluor780 (both Thermo Fisher Scientific) was used according to manufacturer’s protocol with 0.05µl or 0.025 µl reactive dye per sample. For intracellular cytokine staining, cells were fixed and permeabilized with the BD Cytofix/Cytoperm^TM^ kit (BD Biosciences) after cell-surface staining according to manufacturer’s instructions, followed by an incubation step with mAbs against IFN-γ. Single-color samples were prepared for automatic compensation. FCM analyses were performed on a FACSCanto II equipped with a high throughput sampler (BD Biosciences). At least 2 x 10^5^ lymphocytes were recorded per sample. Data was analyzed with FACSDiva software (Version 8.0., BD Biosciences) and FlowJo software (Version 10.2, Tree Star, Ashland, OR).

For all experiments a uniform gating strategy was applied (**Supp Figure 1**). Lymphocytes were gated according to their light scatter properties and potential doublet cells were excluded by a FSC-A/FSC-H gate, followed by gating of Near-IR^-^ or VDeFluor780^-^ cells to exclude dead cells (**Supp Figure 1A**). Cells with a high auto fluorescent signal were excluded from further analyses by using a bandpass filter 510/50 nm (**Supp Figure 1C**); such cells were not observed in PBMC (**Supp Figure 1B**). NKp44 gates were set according to FMO controls containing all other markers except the NKp44 staining, for which only the secondary Ab was applied (**Supp Figures 1B, C**, **Figure 2**).

### Statistical Analysis

Data were analyzed for statistical significance by SPSS^®^ (SPSS Statistics Version 20.0, IBM Corp., Armonk, NY). Obtained values were tested for normal distribution by the Kolmogorov-Smirnov test or Shapiro-Wilk test if *n<4*. Where required, data sets were subjected to log-transformation to meet the condition of normality. Data sets that met the requirement of normal distribution were analyzed by paired two-tailed Student’s t-test for two groups or ANOVA for repeated measurements with Bonferroni correction as *post-hoc* analysis if more than two groups were compared. Where necessary, a Greenhouse-Geisser correction was applied when assumption of sphericity was not given. Data sets that did not show normal distribution were analyzed by Wilcoxon signed-rank test for the comparison of two groups and Friedman test for three or more groups. Levels of significance were defined as: *p ≤ 0.05* (indicated by *), *p ≤ 0.01* (indicated by **) and *p ≤ 0.001* (indicated by ***). Graphs were prepared using Graph Pad Prism V5.04 (GraphPad Software, San Diego, CA, USA).

## Results

### Characterization of NKp44 Expression on Porcine PBMC

**Figure 1:**
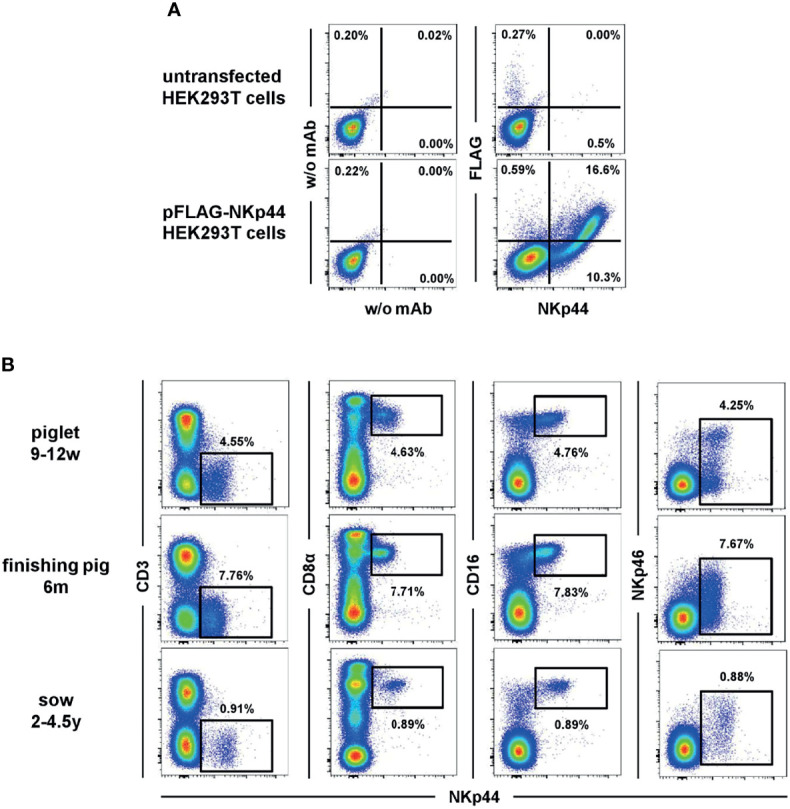
Binding of anti-NKp44 clone 54-1 mAb. **(A)** HEK293T cells were transiently transfected with a mammalian expression vector containing the sequence of porcine NKp44 with an N-terminal FLAG tag. Cells were stained with the anti-NKp44 mAb clone 54-1 and an anti-FLAG mAb. Unstained (w/o mAb) as well as untransfected HEK293T cells served as controls. **(B)** Co-expression of NKp44 with NK-cell related markers on peripheral blood lymphocytes in pigs. Expression of NKp44 was tested by flow cytometry in pigs of three different age groups: 9-12 week old piglets (upper row), 6 month old finishing pigs (middle row) and 2-4.5 year old sows (bottom row). Co-expression of NKp44 with CD3, CD8α, CD16 and NKp46 was analyzed. Results are shown for one representative animal for each age group with frequencies of NKp44^+^ cells indicated.

The first screens of anti-NKp44 hybridoma supernatant revealed positive reactivity with the immunogen, but limited reactivity with porcine lymphocytes in PBMC, spleen and lymph node. Indeed, just one of the hybridomas (clone 54-1) exhibited positive reactivity on porcine lymphocytes. Based on human NK cell data, PBMC were stimulated with IL-2 and IL-15; this resulted in upregulation of staining from 3 to 9% NKp44^+^ PBMC (data not shown). Once these results were verified the anti-NKp44 clone 54-1 was shared with the Vienna lab for more detailed analyses. The anti-porcine NKp44 was tested on FLAG tagged, recombinant NKp44 protein expressed by transiently transfected HEK293T cells (**Figure 1A**). A clear co-staining of the 54-1 mAb clone and the anti-FLAG mAb was visible on transfected HEK293T cells compared to non-transfected cells, confirming specificity of the mAb clone to porcine NKp44.

In a next step, the anti-NKp44 mAb was tested on porcine PBMC *ex vivo*. A distinct population of NKp44^+^ lymphocytes could be detected (**Figure 1B**), notably without stimulation of cells; that is in vast contrast to literature on human NKp44 (7, 9, 10). This *ex vivo* NKp44 expression was observed in animals of different age groups (**Figure 1B**), from piglets (9-12 weeks), finishing pigs (6 month) and sows (2-4.5 years). The NKp44^+^ lymphocytes showed an NK-cell phenotype, as they co-expressed CD8α as well as CD16 and lack of CD3 expression (**Figure 1B**). Notably, a substantial part of the NKp44^+^ cells co-expressed NKp46 (**Figure 1B**, last column). Additionally, a small subset of CD3^+^NKp44^+^ cells could be detected in analyzed animals (**Figure 1B** and **Supp Figure 2**), similar to the previously described NKp46^+^ non-conventional T cells (31). With few exceptions, this CD3^+^NKp44^+^ population accounted for less than 1% of total PBL (**Supp Figure 2**).

**Figure 2:**
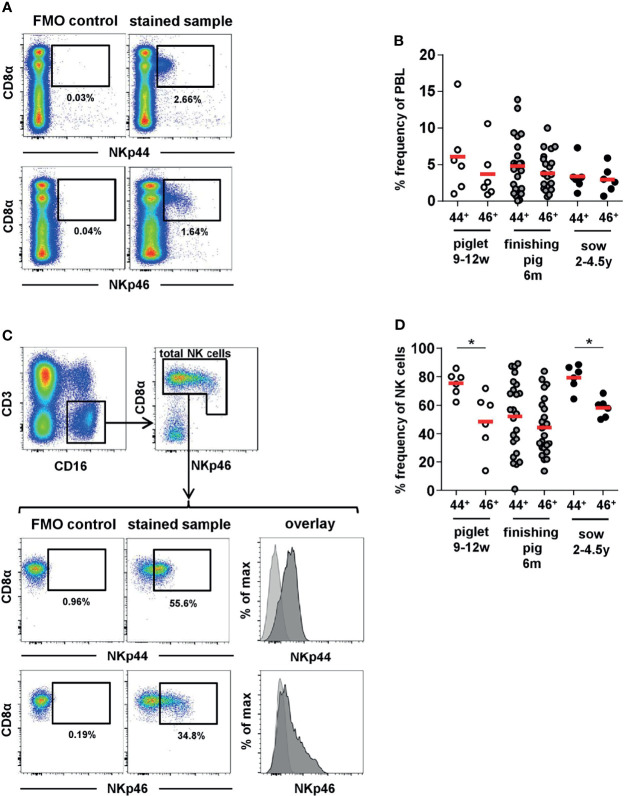
Frequencies of NKp44 and NKp46 expressing cells in porcine blood. Porcine PBMC were tested for the expression of NKp44 and NKp46 by flow cytometry in piglets (*n=6*, open cycles), fattening pigs (*n=24*, grey cycles) and sows (*n=6*, black cycles). **(A)** The frequencies of NKp44^+^ and NKp46^+^ cells within total lymphocytes were analyzed. Graphs show a representative example for a fattening pig. The gates for NKp44 and NKp46 were set according to FMO controls for the distinct markers. **(B)** Frequencies of NKp44^+^ and NKp46^+^ cells within total lymphocytes with mean and standard deviations were as follows: piglets - NKp44: 6.1% ± 5.4, NKp46: 3.7% ± 3.7; finishing pigs - NKp44: 4.8% ± 3.8, NKp46: 3.8% ± 2.5; sows - NKp44: 3.4% ± 2.1, NKp46: 3.0% ± 1.8. One of the animals of the piglet age group showed particularly high frequencies of NKp44^+^ (16.0%) and NKp46^+^ (10.6%) lymphocytes. This was the result of the elevated number of total NK cells in this animal (15.9%). **(C)** The frequencies of NKp44^+^ and NKp46^+^ cells within total porcine NK cells were analyzed. Therefore, lymphocytes were gated on CD3^-^CD16^+^ cells and total NK cells were gated according to their CD8α and NKp46 expression. Graphs show a representative example for a fattening pig, including FMO controls for NKp44 and NKp46. The histograms show the overlay of the FMO control (light grey) and the stained sample (dark grey). **(D)** Frequencies NKp44^+^ and NKp46^+^ cells within total porcine NK cells with mean and standard deviations were as follows: piglets – NKp44: 75.4% ± 8.4, NKp46: 48.7% ± 21.0; finishing pigs - NKp44: 51.2% ± 26.0, NKp46: 44.7% ± 20.2; sows - NKp44: 79.5% ± 8.9, NKp46: 58.3% ± 6.7. Means are indicated by red bars. Significant differences between NKp44 and NKp46 within the different age groups are indicated (*p ≤ 0.05).

Within total lymphocytes, slightly higher frequencies of NKp44^+^ cells compared to NKp46^+^ cells were detected in piglets (**Figures 2A, B**, open cycles). Less obvious differences were observed in finishing pigs (**Figures 2A, B**, grey circles) as well as in sows (**Figures 2A, B**, black circles). Nevertheless, overall higher frequencies of NKp44 compared to NKp46 could be observed for all three age groups, represented by the ratios of NKp44^+^ to NKp46^+^ cells within individual animals (**Supp Figure 3A**). In piglets, nearly double the amounts of NKp44^+^ cells compared to NKp46^+^ were found (open circles) while in sows all animals had a NKp44:NKp46 ratio around 1, indicating nearly identical number of cells expressing the two molecules (black circles). In finishing pigs, 15 out of the 24 animals analyzed had a NKp44:NKp46 ratio ≥1, while 9 animals even had lower numbers of NKp44^+^ cells compared to NKp46^+^ (grey circles).

In a next step, the frequencies of NKp44^+^ and NKp46^+^ cells within total NK cells were analyzed. For this purpose, live lymphocytes were further gated on CD3^-^CD16^+^ cells and total NK cells were identified by their CD8α and NKp46 expression (**Figure 2C**). In piglets the majority of NK cells expressed NKp44, compared to fewer expressing NKp46 (**Figure 2D**, open circles), NKp44^+^ nearly doubling the NKp46^+^ cells as observed for total PBL (**Supp Figure 3B**, open circles). In general, the frequencies of NKp44 expressing NK cells was homogeneous among piglets, while a more heterogeneous picture was observed for NKp46, as previously reported (29). In finishing pigs, an even higher variability in the frequencies of both markers was observed (**Figure 2D** + **Supp Figure 3B**, grey circles). Indeed, one of the 24 animals analyzed had no detectable expression of NKp44. Sows showed homogeneous frequencies of both markers on NK cells (**Figure 2D**, black circles). The majority of sow NK cells expressed NKp44 with slightly reduced levels of NKp46^+^ cells, resulting in a NKp44:NKp46 ratio of ≥1 for all animals (**Supp Figure 3B**, black circles). Overall, the results indicate that the vast majority of NK cells in the pig already exhibit baseline expression of NKp44 *ex vivo* and that this member of the NCR receptor family is more abundant than NKp46 for pigs.

### Distribution of NKp44 and NKp46 Within Total NK Cells in Blood and Organs

**Figure 3:**
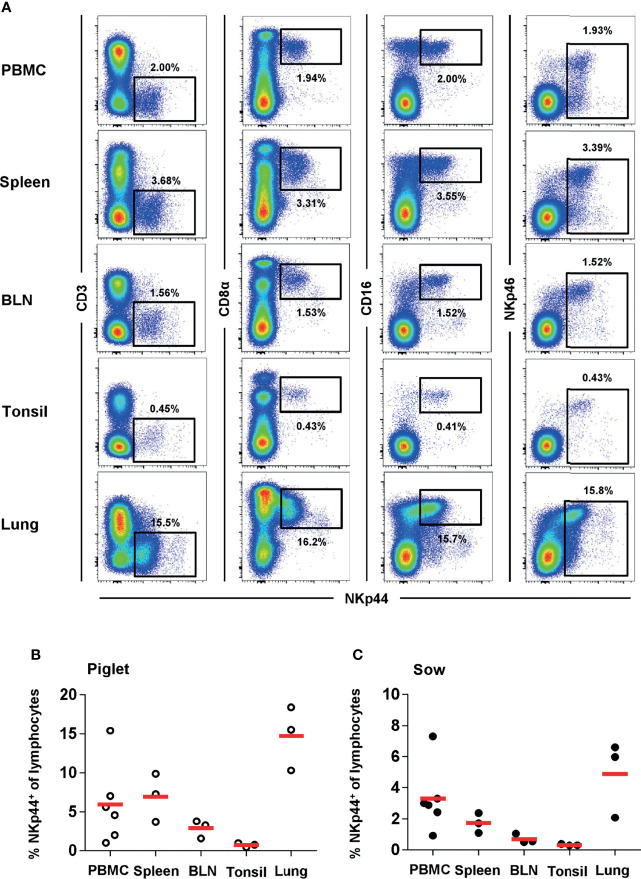
NKp44 expression in cells from blood and organs of 9-12 week old piglets and 2-4.5 year old sows. Expression of NKp44 was analyzed on lymphocytes isolated from blood, spleen, bronchial lymph node (BLN), tonsil and lung from 9-12 week old piglets and 2-4.5 year old sows by FCM. **(A)** Co-expression of NKp44 with CD3, CD8α, CD16 and NKp46 was analyzed. Results are shown for one representative animal (piglet) with frequencies of NKp44^+^ cells indicated. **(B)** Frequencies of NKp44^+^ cells within total lymphocytes of all piglets (PBMC: *n=6*, organs: *n=3*) were analyzed. Frequencies of cell populations with mean and standard deviations were as follows: PBMC: 7.3% ± 7.1, spleen: 6.9% ± 3.1, BLN: 2.9% ± 1.2, tonsil: 0.7% ± 0.3, lung: 14.7% ± 4.1. **(C)** Frequencies of NKp44^+^ cells within total lymphocytes of all sows (PBMC: *n=6*, organs: *n=3*) were analyzed. Frequencies of cell populations with mean and standard deviations were as follows: PBMC: 2.1% ± 1.0, spleen: 1.7% ± 0.6, BLN: 0.7% ± 0.3, tonsil: 0.3% ± 0.1, lung: 4.9% ± 2.5. Means are indicated by red bars.

The unexpected NKp44 expression on NK cells from blood *ex vivo* and the high frequencies of NKp44^+^ cells led to the investigation of different lymphatic and non-lymphatic organs of piglets and sows for this marker. Indeed, comparable to blood, we could identify a distinct population of NKp44^+^ lymphocytes in spleen, bronchial lymph node (BLN), tonsils and lung that share the CD3^-^CD8α^+^CD16^+^NKp46^-/+^ NK-cell phenotype in piglets (**Figures 3A, B**) and sows (**Figure 3C** and **Supp Figure 4**). For both age groups, the percentage of NKp44 cells were more abundant in PBMC and spleen compared to BLN and tonsils. The highest frequencies of NKp44^+^ and NKp46^+^ cells were found in the non-lymphatic organ, the lung.

**Figure 4:**
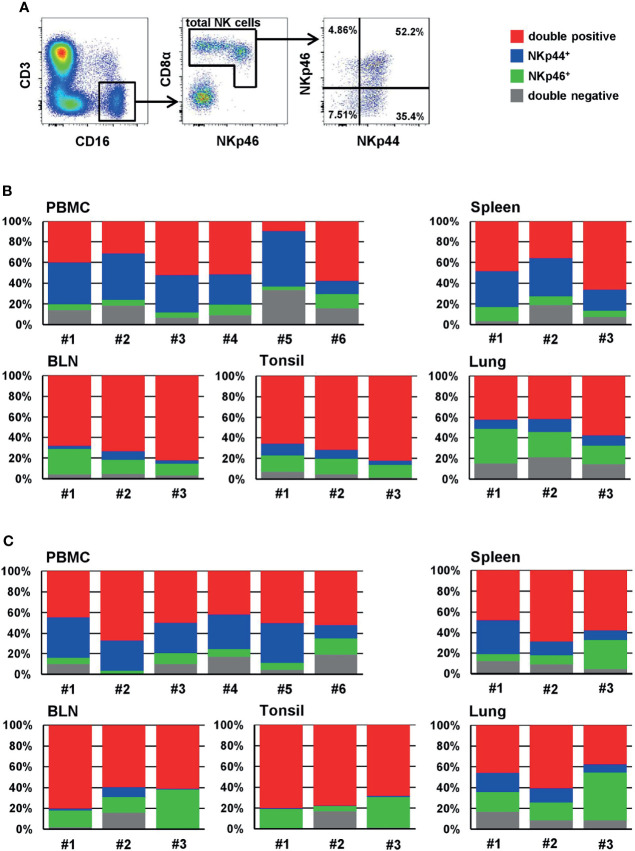
Frequencies of NKp44/NKp46 defined phenotypes of NK cells of blood and organs. **(A)** For analyzing the frequencies of NKp44 and NKp46 expression within NK cells, live lymphocytes were further gated on CD3^-^CD16^+^ cells. In a next step, total NK cells were gated according to their CD8α and NKp46 expression and the frequencies of NKp44 single-positive, NKp46 single-positive as well as NKp44/NKp46 double positive and double negative cells were analyzed. The gating strategy is shown for PBMC of one animal (piglet) and is representative for all animals and organs analyzed. Stacked bar charts show the frequencies of double positive (red), single NKp44^+^ (blue), single NKp46^+^ (green) and double negative cells (grey) within total NK cells of individual animals in PBMC and organs for **(B)** 9-12 week old piglets (PBMC: *n=6*, organs: *n=3*) and **(C)** 2-4.5 year old sows (PBMC: *n=6*, organs: *n=3*). Frequencies of cell populations with mean and standard deviations were as follows: **(B)** PBMC - double-positive: 40.6% ± 17.8, NKp44^+^: 35.8% ± 14.1, NKp46^+^: 7.2 % ± 3.8, double-negative: 16.4 % ± 9.3. Spleen - double-positive: 47.1 % ± 16.2, NKp44^+^: 28.3% ± 8.3; NKp46^+^: 8.3% ± 2.6, double-negative: 16.0% ± 7.4. BLN - double-positive: 74.8 % ± 7.1, NKp44^+^: 4.6% ± 2.9; NKp46^+^: 16.4% ± 7.2, double-negative: 4.2% ± 0.6. Tonsil: - double-positive: 73.5 % ± 8.2, NKp44^+^: 7.7% ± 3.6; NKp46^+^: 14.3% ± 1.7, double-negative: 4.5% ± 2.8. Lung: - double-positive: 47.2 % ± 9.2, NKp44^+^: 10.4% ± 1.9; NKp46^+^: 25.4% ± 8.0, double-negative: 16.9% ± 3.6. **(C)** PBMC - double-positive: 50.5% ± 7.4, NKp44^+^: 29.6% ± 10.0, NKp46^+^: 8.1 % ± 4.6, double-negative: 11.9 % ± 5.3. Spleen - double-positive: 58.4 % ± 10.4, NKp44^+^: 18.3% ± 12.7; NKp46^+^: 14.6% ± 11.9, double-negative: 8.6% ± 3.8. BLN - double-positive: 67.2 % ± 11.5, NKp44^+^: 3.8% ± 4.8; NKp46^+^: 22.8% ± 12.6, double-negative: 6.2% ± 8.4. Tonsil: - double-positive: 75.7 % ± 12.3, NKp44^+^: 0.5% ± 0.3; NKp46^+^: 17.5% ± 12.3, double-negative: 6.4% ± 9.2. Lung: - double-positive: 48.1 % ± 11.8, NKp44^+^: 13.3% ± 5.4; NKp46^+^: 27.1% ± 16.1, double-negative: 11.5% ± 4.9.

Next, we analyzed the distribution of NKp44 and NKp46 expression within CD3^-^CD16^+^ total NK cells in blood and organs of piglets (**Figures 4A, B**) and sows (**Figure 4C**) in more detail to identify NKp44/NKp46- double-positive cells (red), single positive cells (NKp44: blue, NKp46: green) as well as NK cells expressing neither of the two receptors (grey). Among PBMC of piglets, double-positive (**Figure 4B**, red) and NKp44 single-positive cells (blue) constituted the largest proportion of total NK cells, with approximately 40% each. A minor proportion was expressing only NKp46 (green). In all animals a small population of total NK cells in blood was not expressing either of the two receptors (grey). A similar distribution was observed in spleen. A different mixture of phenotypes was observed in BLN and tonsils; for both organs, the vast majority of NK cells expressed both receptors, comprising over 70% (red). Fewer single NKp44^+^ cells (blue) than single NKp46^+^ cells (green) were found in both organs and only a minority of cells did not express either of the receptors (grey). In the lung, half of all NK cells expressed both markers (red), followed by single NKp46^+^ (green), double-negative cells (grey), and a minor fraction of single NKp44^+^ cells (blue).

Comparable to the data from piglets, similar distributions of NKp44 and NKp46 within CD3^-^CD16^+^ NK cells were found in samples collected from sows (**Figure 4C**). In PBMC and lung half of the NK cells expressed both receptors (red). While in PBMC single NKp44^+^ cells (blue) were more abundant than single NKp46^+^ cells (green), the expression pattern of the two populations was reversed in lung. In both organs a minor fraction of up to 10% was double-negative (grey). More than half of splenic NK cells expressed both markers (red), while a more heterogeneous picture was seen for single NKp44^+^ (blue) and single NKp46^+^ (green) cells between the different animals. Nevertheless, on an average value, similar levels of both markers were found. Slightly fewer double-negative NK cells were found in the spleen (grey). The vast majority of NK cells in BLN and tonsils expressed both markers, again with approximately 70% (red). The next most frequent population in both organs was single NKp46^+^ cells (green); very few double-negative cells were found (grey) and nearly no NKp44^+^ cells, that was especially obvious in the tonsils (blue).

Additionally, we analyzed the four subsets in the PBMC samples of the finishing pigs (**Supp Figure 5**). As results in **Figure 2B** already indicated, very heterogeneous phenotypes between animals were seen.

### Induction of NKp44 Expression on Porcine and Human NK Cells

**Figure 5:**
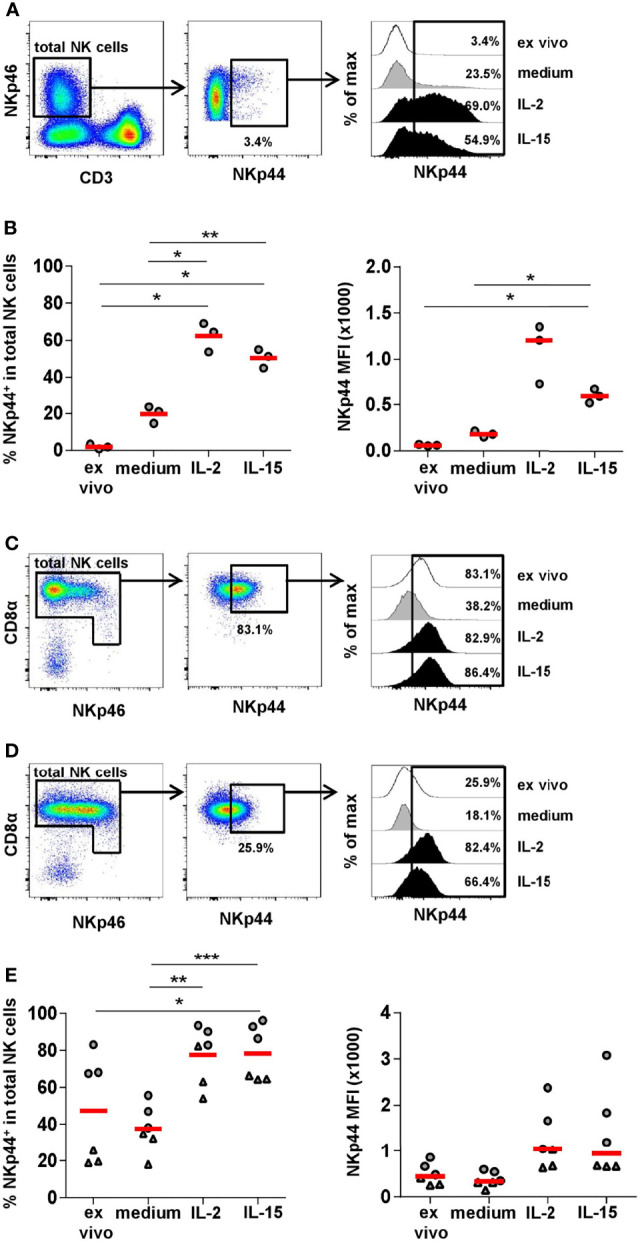
NKp44 expression on human and porcine NK cells after *in vitro* stimulation. Human PBMC from healthy adult donors (*n=3*) and porcine PBMC from 6-month old finishing pigs (*n=6*) were stimulated *in vitro* for 7 days with IL-2 (50 ng/ml) or IL-15 (50 ng/ml). Cells cultured in medium alone served as the control. On day 7, cells were analyzed for NKp44 expression by flow cytometry. Non-cultivated cells (*ex vivo*) were stained in parallel. **(A)** For analyzing NKp44 induction in humans, live lymphocytes were gated on CD3^-^NKp46^+^ cells. Graphs are from one representative donor under *ex vivo* conditions. Histograms on the right show NKp44 expression for all four conditions. **(B)** Frequencies of NKp44^+^ cells within total NK cells for all donors are shown in the graph on the left for all four conditions. Median fluorescence intensities (MFIs) are shown in the graph on the right. **(C)** Frequencies of NKp44^+^ cells with mean and standard deviations of NK cells within cultured PBMCs were as follows: *ex vivo*: 1.9% ± 1.3, medium: 19.7% ± 4.6, IL-2: 62.3% ± 7.9, IL-15: 50.3% ± 5.1. MFIs with median and standard deviations were as follows: *ex vivo*: 59.8 ± 7.9, medium: 179.0 ± 32.6, IL-2: 1205.0 ± 322.5, IL-15: 598.0 ± 75.0. **(C+D)** For analyzing NKp44 induction in pigs, CD3^-^CD16^+^ cells were gated as shown in **Figure 4**. In a next step, total NK cells were gated according to their CD8α and NKp46 expression and the frequency of NKp44^+^ cells was analyzed. Graphs are from two representative animals (**C**: *ex vivo* high NKp44 expression, **D**: *ex vivo* low NKp44 expression). Histograms on the right show NKp44 expression for all four conditions of the respective animals. **(E)** Frequencies of NKp44^+^ cells within total NK cells for all animals are shown in the graph on the left for all four conditions. Median fluorescence intensities (MFIs) are shown in the graph on the right. Frequencies of NKp44^+^ cells with mean and standard deviations were as follows: *ex vivo*: 47.3% ± 28.8, medium: 37.8% ± 12.9, IL-2: 77.7% ± 15.7, IL-15: 78.5% ± 15.0. MFIs with median and standard deviations were as follows: *ex vivo*: 448.5 ± 234.1, medium: 334.0 ± 166.7, IL-2: 1039.0 ± 667.3, IL-15: 940.0 ± 965.1. Animals with high *ex vivo* NKp44 expression are indicated by grey circles, animals with low *ex vivo* NKp44 expression are indicated by grey triangles. Means and medians are indicated by red bars. Significant differences are indicated (*p ≤ 0.05, **p ≤ 0.01, ***p ≤ 0.001).

It is reported that despite lacking *ex vivo* NKp44 expression in human NK cells, high levels can be induced after *in vitro* cytokine stimulation (7, 9, 10). Therefore, we sought to replicate this with human PBMC and compare it to porcine PBMC. Samples from three individual human donors were stimulated for seven days with either IL-2 or IL-15 and NKp44 expression within CD3^-^NKp46^+^ NK cells was compared to unstimulated cells as well as *ex vivo* stained PBMC (representative example in **Figure 5A**). As expected, a vast increase in the percentage of human NKp44^+^ NK cells as well as in NKp44 median fluorescence (MFI) could be observed after IL-2 as well as IL-15 stimulation with approximately 50% of NK cells gaining NKp44 expression compared to nearly non-existent NKp44 expression *ex vivo* (**Figure 5B**). Although not statistically significant, a higher induction of NKp44 was achieved with IL-2 as compared to IL-15. For human PBMC a slight increase in NKp44 expression was seen in medium cultivated cells (**Figure 5B**) compared to the *ex vivo* situation.

To investigate NKp44 induction on porcine NK cells, total PBMC from six individual animals (finishing pigs, 6 month old) were stimulated *in vitro* for seven days with either IL-2 or IL-15, then NKp44 expression within total NK cells was compared to unstimulated cells and to *ex vivo* expression (**Figures 5C–E**). Because we had already observed variable NKp44 frequencies in porcine NK cells, we selected three animals with initial high percentage of NKp44^+^ NK cells (above 60%, representative example in **Figure 5C**), as well as three animals with lower amounts (below 40%, representative example in **Figure 5D**). For all animals we could observe lower NKp44 frequencies, as well as MFIs, in the medium control compared to *ex vivo* analyzed samples (**Figure 5E**). Nevertheless, a clear increase in NKp44 expression was observed after IL-2 or IL-15 stimulation (**Figure 5E**); however, no clear difference was observed between the two cytokine treatments. In those animals exhibiting high frequencies of NKp44^+^ NK cells prior to stimulation, only a maximal 1.4-fold increase was observed after cytokine stimulation. In contrast, in the group of animals initially showing lower NKp44^+^ NK-cell frequencies a 2.5 - 3.5-fold increase was observed after cytokine stimulation. Interestingly, NKp44 MFIs increased to a lesser degree in animals with initial lower NKp44^+^ NK-cell frequencies (1.4 - 2.7-fold) compared to those that already showed higher NKp44^+^ numbers (1.6 - 3.6-fold). No such effect was seen after IL-2 or IL-15 stimulation on CD3^+^NKp44^+^ lymphocyte subsets (**Supp Figure 6**).

### Degranulation of NK Cells Can Be Induced by NKp44 Receptor Crosslinking

**Figure 6:**
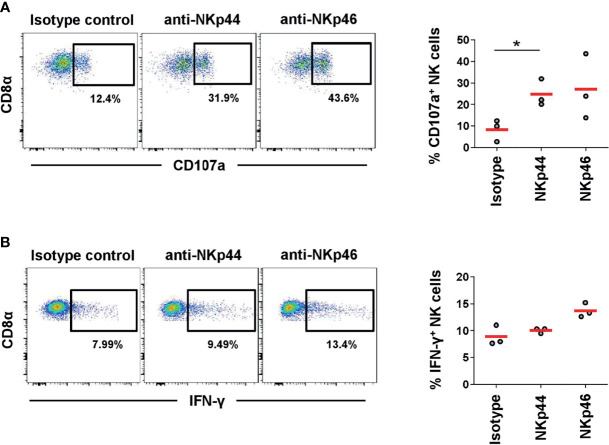
Activation of NK cells by NKp44 receptor triggering. Receptor-mediated degranulation and IFN-γ was assessed by multicolor FCM in response to cross-linking of NKp44 or NKp46 by plate-bound mAbs. Isotype-matched irrelevant antibodies served as control. PBMC from 6-month old finishing pigs (*n=3*) were gated on CD3^-^CD8α^+^CD16^+^. **(A)** Cells were pre-activated with rPoIL-2 and rPoIL-15 for 24 h and receptor-mediated degranulation was assessed by CD107a expression on the cell surface after 4 h incubation with plate-bound mAbs. **(B)** Cells were pre-activated with rPoIL-2 and rPoIL-18 for 24 h and intracellular IFN-γ production was measured after receptor cross-linking for 4 h. Dot-plots show results of one representative animal. Frequencies of CD107a^+^ and IFN-γ^+^ of all three animals analyzed are shown in the graphs on the right. Mean values are represented by red bars. Frequencies of CD107a^+^ cells with mean and standard deviations of NK cells within cultured PBMCs were as follows: isotype control: 8.3% ± 5.1, NKp44: 24.7% ± 6.4, NKp46: 27.1% ± 15.2. Frequencies of IFN-γ^+^ cells with mean and standard deviations of NK cells within cultured PBMCs were as follows: isotype control: 8.9% ± 1.9, NKp44: 10.0% ± 0.5, NKp46: 13.7% ± 1.3. Significant differences are indicated (*p ≤ 0.05, using the Sidak *post-hoc* analysis).

For human NK cells it was shown that crosslinking of NKp44 leads to activation of NK cells (7). Therefore, we investigated degranulation by measuring CD107a expression as well as IFN-γ production after receptor triggering by plate-bound mAbs directed against NKp44 or NKp46. Plates coated with isotype-matched control antibodies served as negative control. A clear 3-fold increase in CD107a^+^ NK cells was observed after NKp44 crosslinking compared to the control (**Figure 6A**). Likewise, the same induction of CD107a was achieved after NKp46 triggering. In contrast, stimulation of NK cells by NKp44 plate-bound mAbs did not lead to induction of IFN-γ production in NK cells (**Figure 6B**). Triggering of NKp46 resulted in a minor 1.5-fold increase in IFN-γ^+^ cells compared to the control (although not statistically significant). Nonetheless, data indicate that NKp44 has an activating function on porcine NK cells.

## Discussion

In human NK cells, the cell surface receptor NKp44 has been linked to NK-cell activation as it is only found on stimulated NK cells (7, 9, 10). Few studies on the phenotype and activation state of porcine NK cells were available previously. In this study, we characterized a newly generated mAb against porcine NKp44 to provide a more detailed phenotypic characterization of porcine NK cells. With the new anti-NKp44 mAb, clone 54-1, NKp44 expression was analyzed on PBL as well as cells from lymphatic and non-lymphatic organs from pigs of different age classes: piglets, finishing pigs and sows. Our results showed that, regardless of the age group, the majority of *ex vivo* NK cells expressed baseline NKp44.

Interestingly, very high numbers of NKp44^+^ NK cells were observed in young animals which lack a fully activated adaptive immune system and must rely on a better armed innate immune cell repertoire. The more heterogeneous expression of NKp44, as well as NKp46, observed in finishing pigs may result from different breeding stocks at each farm which in turn exhibit different activation states of their immune systems that vary with housing conditions. Although most animals used for meat production in Austria belong to the Large White x German Landrace x Pietrain crossbreed, no detailed information was available on the slaughterhouse animals. Therefore, variability in NKp44 as well as NKp46 expression might also be influenced by the genetic background of the animals. Results on the high heterogeneity in NKp46 expression match our previous findings (29). Similar high frequencies of NKp44^+^ NK cells were observed in piglets and sows, although higher frequencies of NKp46^+^ NK cells were detected in the latter. NKp46 expression showed a homogeneous pattern in sows in contrast to piglets and finishing pigs. This is consistent with an age-dependent NK-cell phenotype where the majority of NK cells were expressing both receptors.

For all age groups, the ratios of NKp44 to NKp46 expressing cells in blood were higher in total NK cells compared to total lymphocytes. This indicates, at least in the blood, that more NKp46 than NKp44 expressing non-NK cell lymphocytes must be present. These cells potentially belong to the recently described CD3^+^NKp46^+^ non-conventional T cells, where also NKp44 mRNA was detected (31). Our results indicate a minor population co-expressing CD3 and NKp44 in blood. The frequencies of the CD3^+^NKp44^+^ cells was slightly smaller than that of CD3^+^NKp46^+^ in analyzed animals. Therefore, in contrast to NK cells, non-conventional T cells seem to express higher frequencies of NKp46 compared to NKp44. Furthermore, in contrast to a subset of human pDCs, where expression of NKp44 was observed (22, 23), no such expression was seen on porcine pDCs *ex vivo*. On the contrary, the vast majority of porcine pDCs express NKp46, highlighting the unique expression pattern of NCRs on porcine cells (manuscript in preparation). To date, there is no data on expression of pig NKp30, the third NCR family member, due to the lack of species-specific mAbs directed against this marker; however, NKp30 mRNA was detected in porcine NK cells (30). Overall, there is no pan-marker available for porcine NK cells. Thus, identification of porcine NK cells and the respective NK-cell subpopulations, as defined by multi-color FCM staining, is still a challenging task.

Our results show that NKp44^+^ cells were observed *ex vivo* not only in blood, but also in all organs analyzed. Although, data obtained from piglets and sows were comparable, differences in the frequencies of NK-cell subsets between the organs were observed. In piglets and sows, NKp44^+^NKp46^+^ double-positive cells were the most abundant subset within total NK cells in all organs. In spleen, more NKp44^+^ than NKp46^+^ cells were found, while the opposite was found in BLN, tonsils and lung.

Next, we investigated NKp44 induction in pig cells inasmuch as this has been demonstrated in human and non-human primate NK cells (6, 7, 9, 10) and confirmed in our studies. With the pre-existing *ex vivo* expression of NKp44, we probed the differences in animals with an initial high versus low NKp44 expression. For both groups, induction of NKp44 was observed after IL-2 or IL-15 stimulation with no substantial differences between cytokines. As estimated from the initial *ex vivo* NKp44 expression, there was a greater potential for increasing the frequency of NKp44^+^ NK cells in animals with an initial lower NKp44^+^ population. Nevertheless, animals with an initially higher NKp44 expression showed a greater potential to increase the expression MFI of the marker on a cellular level. In contrast to stimulated human γδ T cells (24), no obvious induction of NKp44 could be observed on porcine T-cell subsets compared to medium cultivated cells. Only slightly increased frequencies were observed in CD3^+^TCRγδ^+^ cells compared to the *ex vivo* analyzed samples.

This study shows that the NCR family members, NKp44 and NKp46, are differentially expressed in the pig compared to other species. All NK cells in humans express NKp46 which has been described as reliable marker for NK-cell identification in combination with a CD3^-^ phenotype in all mammalian species (5). In humans, NKp44 expression can be detected on the cell surface only after stimulation (7, 9, 10). Although variability in the expression of NKp44 on porcine NK cells was observed in most animals, the majority was expressing NKp44 already *ex vivo* in high frequencies. Moreover, the proportion of NKp44^+^ NK cells was even higher than the proportion of NKp46^+^ NK cells. This makes the NK-cell phenotypes unique in pigs.

Out data showed an activating function of NKp44, as receptor triggering led to degranulation of NK cells comparable to what was shown for NKp46 and CD16 (30, 31). For human NK cells it was reported that in addition to inducing cytolytic function, NKp44 crosslinking also leads to release of cytokines like IFN-γ and TNF-α (7). We only observed a minor induction of IFN-γ after NKp46 crosslinking that is in accordance with previous results where we could show that stimulation with cytokines was more effective than NKp46 triggering in porcine NK cells (31). No obvious IFN-γ production was observed in porcine NK cells after NKp44 crosslinking. This might indicate that for distinct functional pathways stimulation of one activating receptor might not be sufficient and other co-stimulatory signals might be needed.

In the scope of future studies, it will be interesting to investigate the four NKp44/NKp46 defined NK-cell subsets in regards to other activation markers like CD25 and CD69. For the latter recently the generation of a porcine-specific mAb was described, allowing for the analysis in flow cytometry (37). The TNF receptor family member CD27 was a helpful NK-cell marker in the human and mouse system, dividing NK cells into functionally distinct subsets (38, 39). Likewise, CD27 was shown to be differentially expressed on porcine NKp46-defined NK-cell subsets (30) and thus will be interesting to be analyzed in the context of NKp44 expression. For the pig it was shown previously that the NKp46^high^ NK cells are in a more activated state compared to the other NK-cell subsets (30). The detailed analysis of NKp44/NKp46 defined NK-cell subsets in the context of cytokine production and cytolytic potential after *in vitro* stimulation with e.g. cytokines will therefore contribute to an even more detailed understanding on the diversity of porcine NK-cell biology and will be addressed in future studies.

The availability of mAbs against porcine NKp44, and these first results on NKp44 expression on porcine NK cells, will be vital for future studies with swine. For example, Forberg et al. (35) demonstrated the role of NK cells in influenza infection, although only NKp46 was analyzed. Thus, a potential role of NKp44^+^ cells might be of equal importance since it was shown in humans that NKp44 can bind to influenza hemagglutinins comparably to NKp46 (16, 17). Human NKp44 plays a role in decidual NK cells, as the receptor is constitutively expressed by a specialized subset of non-lymphatic NK cells. This potentially implicates a role for NKp44 during placentation (40). Human decidual NK cells seem to be in a more activated state due to NKp44 expression, although they have regulatory functions, as they are less cytotoxic and produce IL-10 and TGF-β. Therefore, it will be interesting to investigate the role of NKp44 in porcine pregnancy and especially in reproductive disorders or disease e.g., PRRS (porcine reproductive and respiratory syndrome) virus infections. A potential role of NK cells was already demonstrated for PRRS, although increasing as well as decreasing NK-cell numbers were shown dependent on PRRS viral strains (41, 42).

In summary, the availability of a porcine-specific anti-NKp44 mAb (clone 54-1) has allowed us to study the unique phenotype of porcine NK cells in more detail and will greatly aid in elucidating the differentiation and function of NK cells in swine immunity under steady state and disease conditions.

## Data Availability Statement

The raw data supporting the conclusions of this article will be made available by the authors, without undue reservation.

## Author Contributions

PB, DZ, and JL cloned pig NKp44. BW, SB, and LN prepared the mouse immunogen and performed the hybridoma fusions. AC, PB, and JL performed the early mAb anti-NKp44 analyses. KM, WG, AS, MS, and KD planned and/or performed NKp44/NKp46 co-expression studies and stimulation assays. KM and KD performed NKp44 re-cloning. KM, AC, JL, WG, and AS wrote the early drafts of the manuscript. All authors reviewed and approved the final manuscript.

## Funding

This work was started as part of the US Veterinary Immune Response Network, supported by USDA NIFA grant #2010-65121-20649, and continued with USDA ARS project 8042-32000-102 funds. The Christian Doppler Laboratory for Optimized Prediction of Vaccination Success in Pigs is supported by Boehringer Ingelheim Vetmedica GmbH. The financial support by the Austrian Federal Ministry for Digital and Economic Affairs, the National Foundation for Research, Technology and Development and the Christian Doppler Research Association is gratefully acknowledged.

## Conflict of Interest

The authors declare that the research was conducted in the absence of any commercial or financial relationships that could be construed as a potential conflict of interest.

## Publisher’s Note

All claims expressed in this article are solely those of the authors and do not necessarily represent those of their affiliated organizations, or those of the publisher, the editors and the reviewers. Any product that may be evaluated in this article, or claim that may be made by its manufacturer, is not guaranteed or endorsed by the publisher.
