# Supplementary material for: The Natural Cytotoxicity Receptor NKp44 (NCR2, CD336) Is Expressed on the Majority of Porcine NK Cells Ex Vivo Without Stimulation
Source: Front Immunol. 2022 Jan 28;13:767530. doi: 10.3389/fimmu.2022.767530 (PMC8832162; doi:10.3389/fimmu.2022.767530)
Supplement: Supplementary file 1 [file DataSheet_1.pdf]

## *Supplementary Material*

# The Natural Cytotoxicity Receptor NKp44 (NCR2, CD336) is expressed on the majority of porcine NK cells *ex vivo* without stimulation

**Running title:**
Characterization of porcine NKp44 expression

**Authors:**
Kerstin H. Mair[1,3*], Assiatu J. Crossman[2,4*], Bettina Wagner[5], Susanna Babasyan[5], Leela Noronha[5,6], Patricia Boyd[2], Dante Zarlenga[2], Maria Stadler[1], Katinka A. van Dongen[3], Wilhelm Gerner[1,3,7], Armin Saalmüller[1], and Joan K. Lunney[2 #]

**Affiliations:**
[1]Institute of Immunology, Department of Pathobiology, University of Veterinary Medicine Vienna, Vienna, Austria
[2]Animal Parasitic Disease Laboratory, BARC, ARS, USDA, Beltsville, MD 20705, USA
[3]CD Laboratory for Optimized Prediction of Vaccination Success in Pigs, Institute of Immunology, Department of Pathobiology, University of Veterinary Medicine Vienna, Vienna, Austria
[4]Center for Cancer Research, National Cancer Institute, NIH, Building 10, Room 4B36, Bethesda, MD 20892, USA
[5]Department of Population Medicine and Diagnostic Sciences, College of Veterinary Medicine, Cornell University, Ithaca, NY, USA
[6]USDA ARS Arthropod-Borne Animal Diseases Research Unit, ARS, USDA, Manhattan, KS 66502, USA
[7]The Pirbright Institute, Woking, United Kingdom
* These authors have contributed equally to this work and share first authorship

**Correspondence:**
Joan K. Lunney, PhD., APDL, BARC, ARS, USDA, Building 1040, Room 103, BARC-East, Beltsville, MD 20705, USA. PH: 240-645-5536; FAX: 301-504-5306; Email: Joan.Lunney@usda.gov

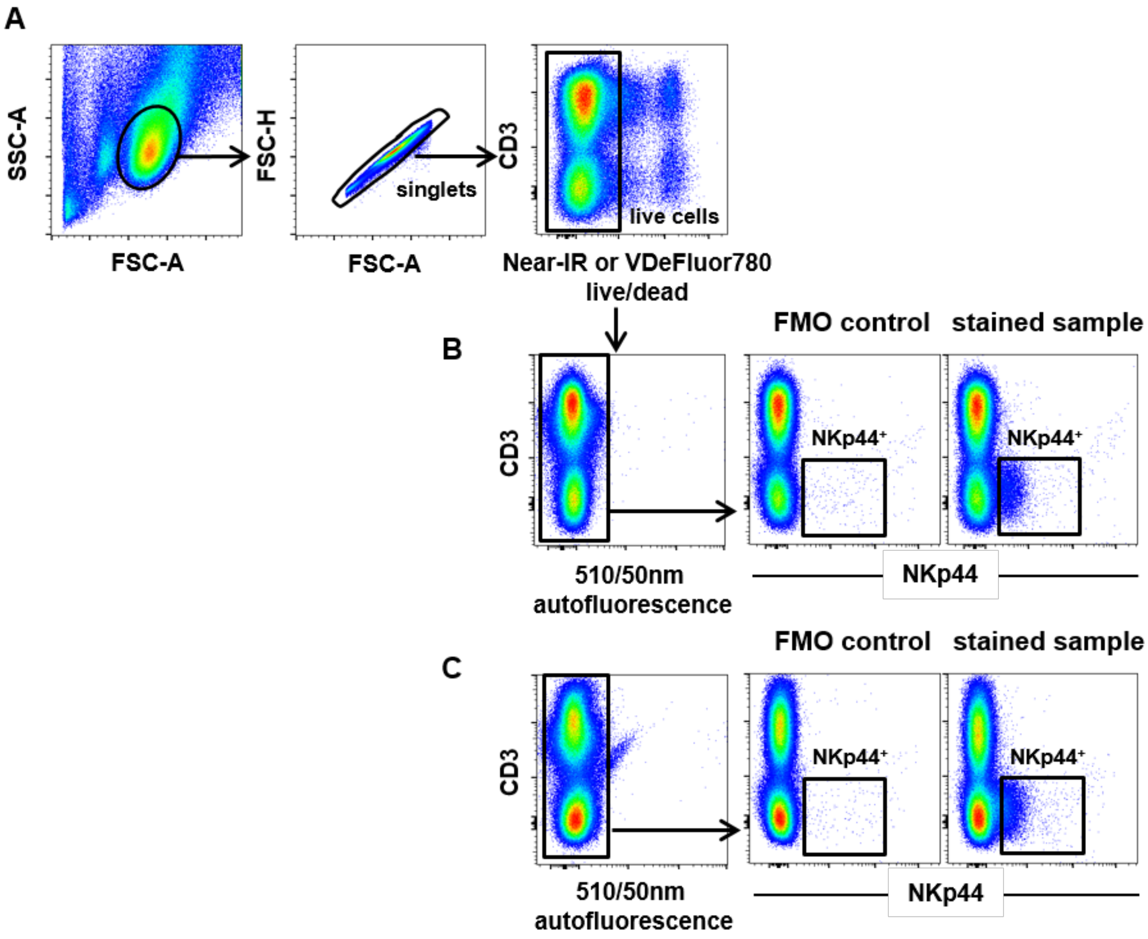


**SUPP Figure 1: Gating strategy for FCM analyses of NKp44 expression. (A)** Lymphocytes of PBMC and all organs analyzed were gated according to their light scatter properties. Potential doublet cells were excluded by a FSC-A/FSC-H gate, followed by gating of Near-IR$^-$ or VDeFluor780$^-$ cells to exclude dead cells. A representative example is shown for PBMC. **(B+C)** Cells with a high auto fluorescent signal were excluded from further analyses by using a bandpass filter 510/50 nm. Such cells were not observed in PBMC **(B)** but were found in lymphatic and non-lymphatic organs (**C**: representative example for spleen). The NKp44 gate was set according to an FMO (fluorescence minus one) control.

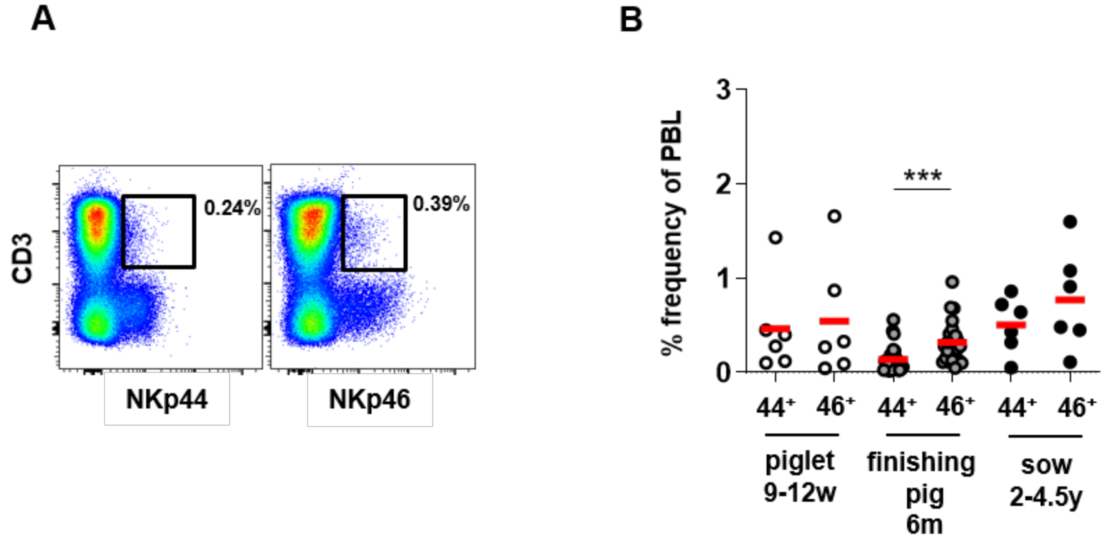


**SUPP Figure 2: Frequencies of NKp44 and NKp46 expressing CD3$^+$ cells in porcine blood.** The frequencies of NKp44$^+$ or NKp46$^+$ cells co-expressing CD3 within total PBL was analyzed. **(A)** Dot plots show one representative example of a finishing pig. Frequencies of double-positive cells are indicated. **(B)** Frequencies of CD3$^+$NKp44$^+$ or CD3$^+$NKp46$^+$ within PBL are shown for piglets (*n=6*, open cycles), fattening pigs (*n=24*, grey cycles) and sows (*n=6*, black cycles). Frequencies of cell populations with mean and standard deviations were as follows: piglets - CD3$^+$NKp44$^+$: 0.46% ± 0.49, CD3$^+$NKp46$^+$: 0.54% ± 0.62; finishing pigs – CD3$^+$NKp44$^+$: 0.14% ± 0.15, CD3$^+$NKp46$^+$: 0.32% ± 0.22; sows – CD3$^+$NKp44$^+$: 0.50% ± 0.30, CD3$^+$NKp46$^+$: 0.77% ± 0.53. Means are indicated by red bars. Significant differences between NKp44 and NKp46 within the different age groups are indicated (*** $p \leq 0.001$).

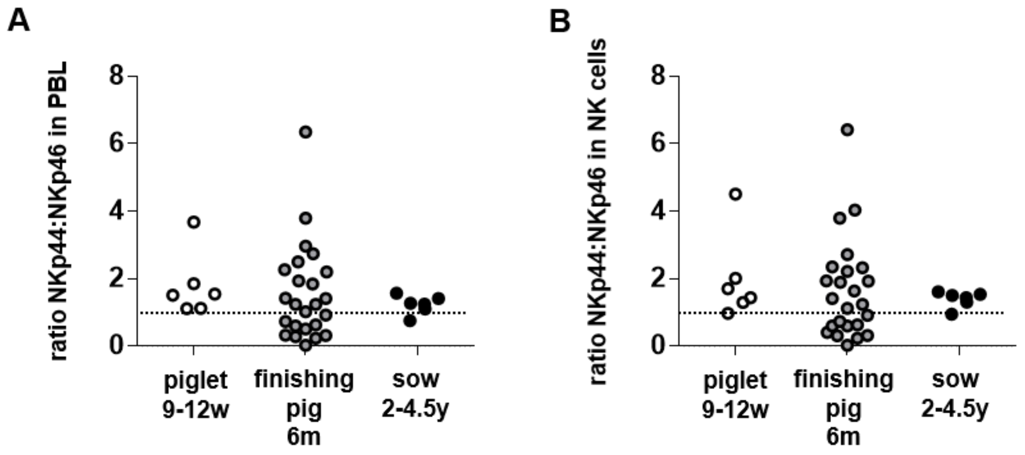


**SUPP Figure 3: Ratios of NKp44 and NKp46 expressing cells in porcine blood.** The ratios of NKp44$^{+}$ to NKp46$^{+}$ cells within total PBL **(A)** and within CD3$^{-}$CD16$^{+}$CD8α$^{+}$ NK cells **(B)** are shown for piglets (*n=6*, open cycles), fattening pigs (*n=24*, grey cycles) and sows (*n=6*, black cycles). Ratios were calculated by dividing the % NKp44$^{+}$ cells by the % NKp46$^{+}$ cells of the respective animal. Black dashed lines represent a ratio of 1 (equal number of NKp44$^{+}$ and NKp46$^{+}$ cells).

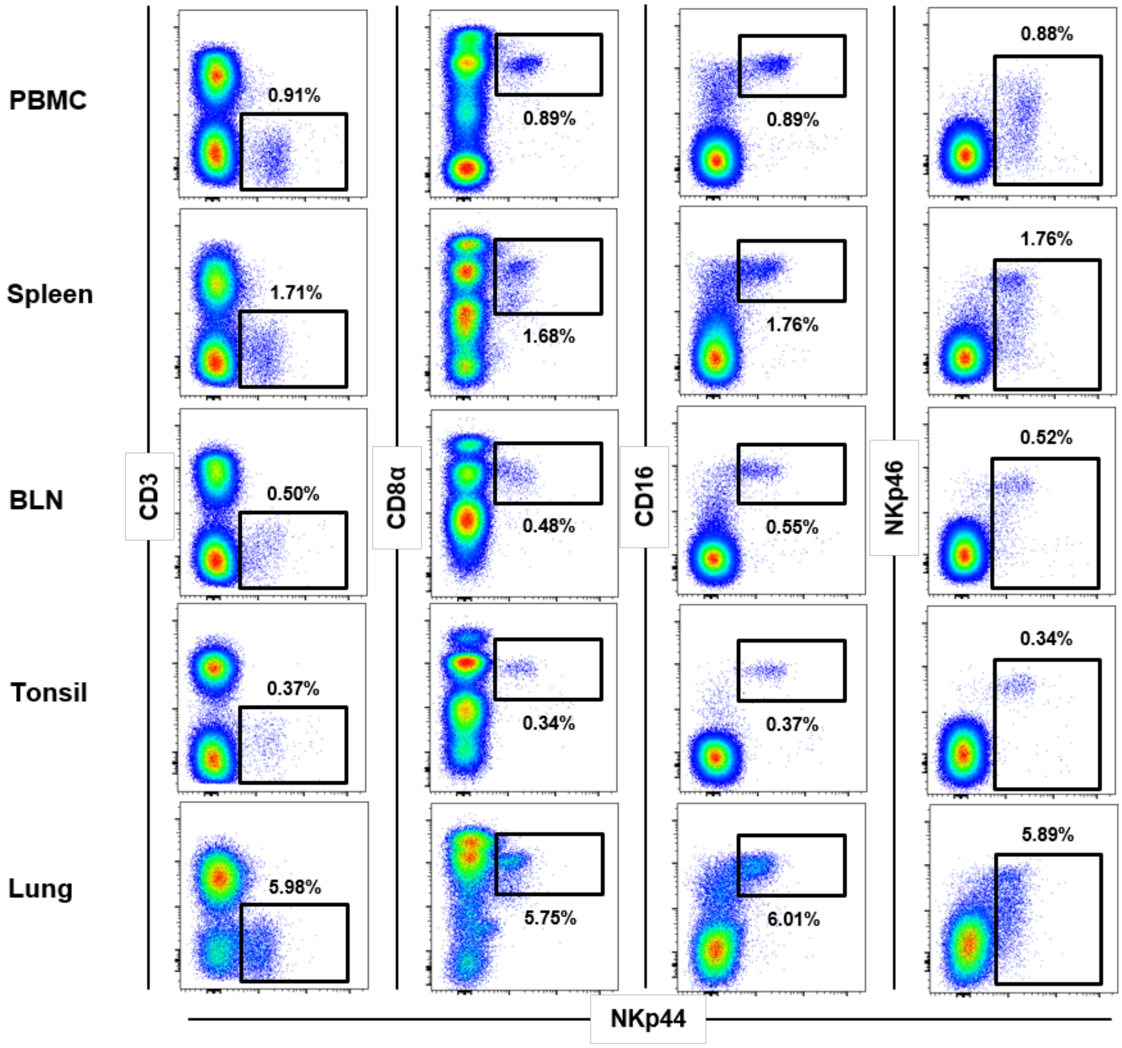


**SUPP Figure 4: NKp44 expression in cells from blood and organs of 2-4.5 year old sows.** Expression of NKp44 was analyzed on lymphocytes isolated from blood, spleen, bronchial lymph node (BLN), tonsil and lung from 2-4.5 year old sows (PBMC: *n=6*, organs: *n=3*) by flow cytometry. Co-expression of NKp44 with CD3, CD8α, CD16 and NKp46 is displayed. Results are shown for one representative animal with frequencies of NKp44$^{+}$ cells indicated.

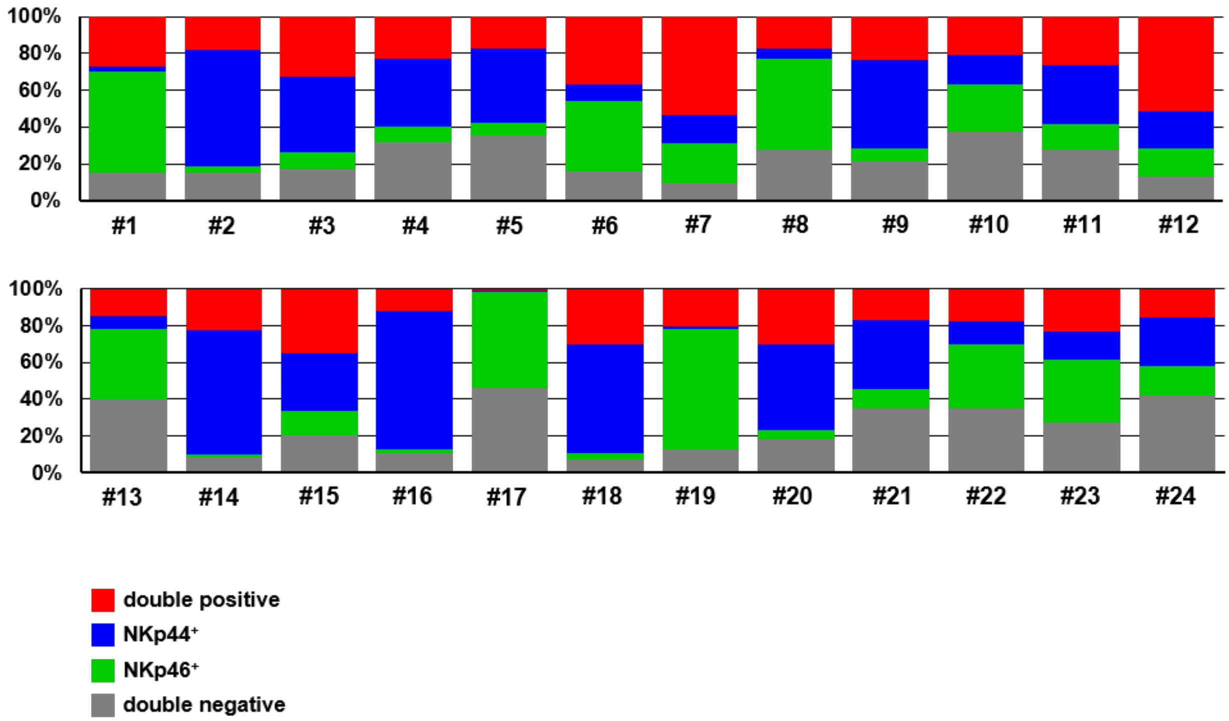


**SUPP Figure 5: NKp44/ NKp46 defined phenotypes of NK cells in blood of 6 months old fattening pigs.** Expression of NKp44 and NKp46 was analyzed on lymphocytes isolated from blood from 6 months old fattening pigs (*n=24*) by flow cytometry. Stacked bar charts show the frequencies of NKp44 and NKp46 double positive (red), single NKp44$^{+}$ (blue), single NKp46$^{+}$ (green) and double negative cells (grey) within total NK cells. For gating of cell populations, the same gating strategy as shown in Figures 4A was used. Frequencies of cell populations with mean and standard deviations were as follows: double-positive: 24.4% ± 11.6, NKp44$^{+}$: 29.8% ± 22.2, NKp46$^{+}$: 22.1% ± 19.1, double-negative: 23.8% ± 11.8.

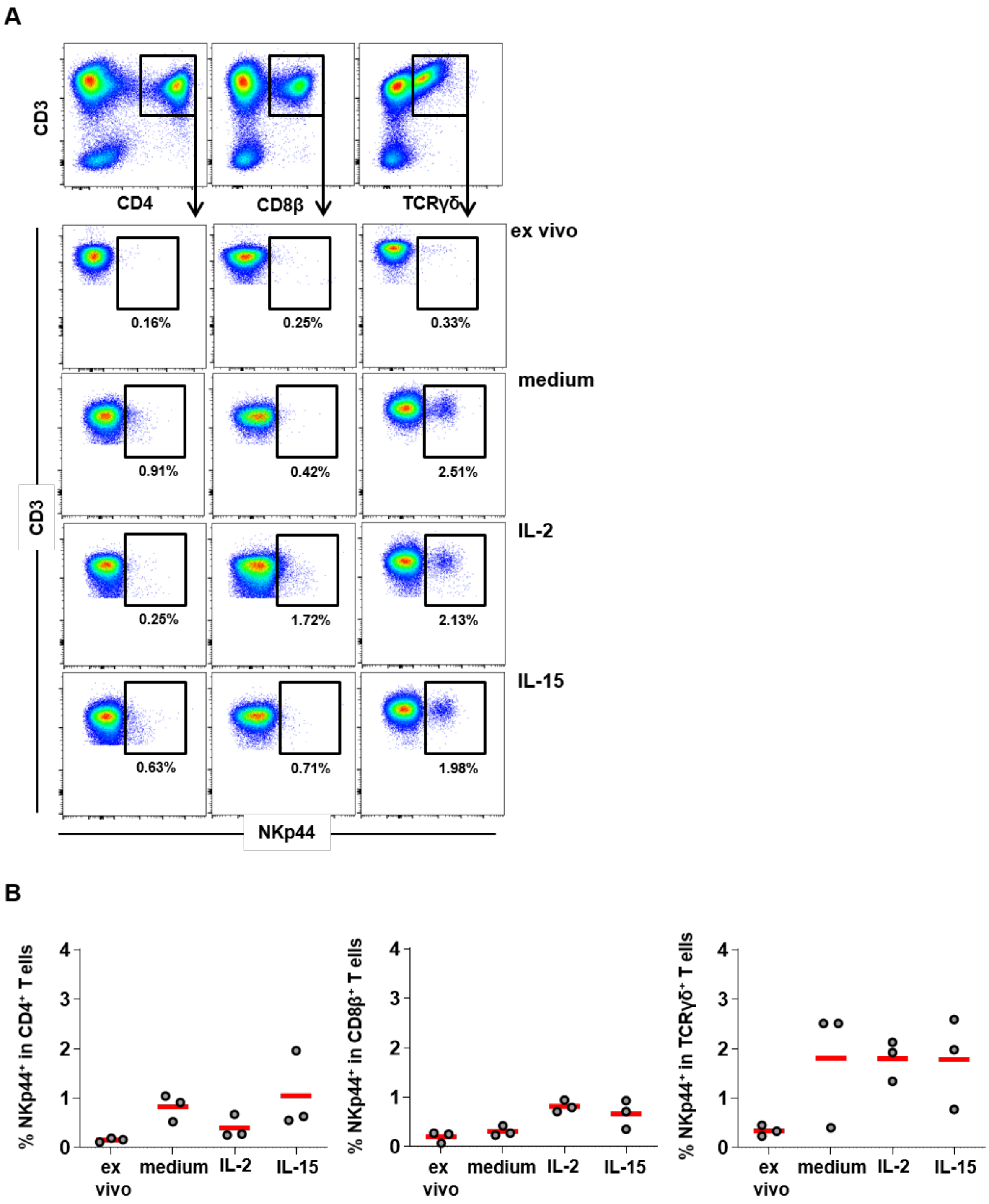


**SUPP Figure 6: NKp44 expression on porcine T-cell subsets after *in vitro* stimulation.** Porcine PBMC from 6-month old finishing pigs (*n=3*) were stimulated *in vitro* for 7 days with IL-2 (50 ng/ml) or IL-15 (50 ng/ml). Cells cultured in medium alone served as the control. On day 7, cells were analyzed for NKp44 expression by flow cytometry. Non-cultivated cells (*ex vivo*) were stained in parallel. **(A)** For analyzing NKp44 induction on porcine T-cell subsets, $CD3^{+}CD4^{+}$, $CD3^{+}CD8\beta^{+}$, or $CD3^{+}TCR\gamma\delta^{+}$ cells were gated. Within these subsets, the frequency of $NKp44^{+}$ cells was analyzed.

Graphs are from one representative animal. **(B)** Frequencies of NKp44$^+$ cells within the distinct T-cell subsets for all animals are shown for all four conditions. Frequencies of NKp44$^+$ cells with mean (red bars) and standard deviations were as follows: CD3$^+$CD4$^+$: *ex vivo*: 0.15% ± 0.04, medium: 0.82% ± 0.27, IL-2: 0.40% ± 0.24, IL-15: 1.05% ± 0.79; CD3$^+$ CD8β$^+$: *ex vivo*: 0.20% ± 0.11, medium: 0.31% ± 0.10, IL-2: 0.81% ± 0.12, IL-15: 0.66% ± 0.29: CD3$^+$ TCRγδ$^+$: *ex vivo*: 0.34% ± 0.11, medium: 1.81% ± 1.22, IL-2: 1.80% ± 0.41, IL-15: 1.78% ± 0.93.
